# Stimulation of tumour growth by wound-derived growth factors

**DOI:** 10.1038/sj.bjc.6690223

**Published:** 1999-03

**Authors:** R Abramovitch, M Marikovsky, G Meir, M Neeman

**Affiliations:** Department of Biological Regulation, The Weizmann Institute of Science, Rehovot, 76100, Israel

**Keywords:** heparin-binding epidermal growth factor, magnetic resonance imaging, C6 glioma spheroid, tumour growth, angiogenesis

## Abstract

The goal of this work was to determine the molecular basis for the induction of tumour vascularization and progression by injury. Magnetic resonance imaging (MRI) studies demonstrated that administration of wound fluid derived from cutaneous injuries in pigs reduced the lag for vascularization and initiation of growth of C6 glioma spheroids, implanted in nude mice, and accelerated tumour doubling time. The former effect can be attributed to the angiogenic capacity of wound fluid as detected in vivo by MRI, and in vitro in promoting endothelial cell proliferation. The latter effect, namely the induced rate of tumour growth, is consistent with the angiogenic activity of wound fluid as well as with the finding that wound fluid was directly mitogenic to the tumour cells, and accelerated growth of C6 glioma in spheroid culture. Of the multiple growth factors present in wound fluid, two key factors, heparin-binding epidermal growth factor (EGF)-like growth factor (HB-EGF) and platelet-derived growth factor (PDGF), were identified as the dominant mitogens for C6 glioma, and inhibition of their activity using specific neutralizing antibodies suppressed the mitogenic effect of wound fluid on DNA synthesis in C6 glioma. This study suggests that the stimulatory effect of injury on tumour progression can possibly be attenuated by therapeutic targeting directed against a limited number of specific growth factors. © 1999 Cancer Research Campaign


British Journal of Cancer (1999) **79**(9/10), 1392–1398
© 1999 Cancer Research Campaign
Article no. bjoc.1998.0223

### Stimulation of tumour growth by wound-derived growth factors

**R Abramovitch, M Marikovsky*†, G Meir and M Neeman**

Department of Biological Regulation, The Weizmann Institute of Science, Rehovot 76100, Israel

**Summary** The goal of this work was to determine the molecular basis for the induction of tumour vascularization and progression by injury. Magnetic resonance imaging (MRI) studies demonstrated that administration of wound fluid derived from cutaneous injuries in pigs reduced the lag for vascularization and initiation of growth of C6 glioma spheroids, implanted in nude mice, and accelerated tumour doubling time. The former effect can be attributed to the angiogenic capacity of wound fluid as detected in vivo by MRI, and in vitro in promoting endothelial cell proliferation. The latter effect, namely the induced rate of tumour growth, is consistent with the angiogenic activity of wound fluid as well as with the finding that wound fluid was directly mitogenic to the tumour cells, and accelerated growth of C6 glioma in spheroid culture. Of the multiple growth factors present in wound fluid, two key factors, heparin-binding epidermal growth factor (EGF)-like growth factor (HB-EGF) and platelet-derived growth factor (PDGF), were identified as the dominant mitogens for C6 glioma, and inhibition of their activity using specific neutralizing antibodies suppressed the mitogenic effect of wound fluid on DNA synthesis in C6 glioma. This study suggests that the stimulatory effect of injury on tumour progression can possibly be attenuated by therapeutic targeting directed against a limited number of specific growth factors.

**Keywords:** heparin-binding epidermal growth factor; magnetic resonance imaging; C6 glioma spheroid; tumour growth; angiogenesis

The increased probability of recurrence and accelerated tumour growth in the location of tissue injury are common clinical complications associated with the invasive procedures frequently used in cancer therapy. The concept that injuries promote tumour development at the injured site was suggested already in 1927 (Deelman, 1927). This concept was extended to show that trauma increased the probability of tumour formation in the injured organ, without affecting the distribution to other sites, by promoting implantation and proliferation of circulating cancer cells (Murthy et al, 1991). The tumours specifically developed in traumatized organs in the injury site, and as the wounds heal their ability to facilitate implantation and/or growth of tumour cells decreases (Murthy et al, 1989). It seems that the same repair processes involved in wound healing also contribute to tumour attachment and growth (Dvorak et al, 1995). We showed previously that injured skin tissue provides a favourable milieu for the neovascularization and growth of C6 glioma spheroids, implanted subcutaneously in nude mice (Abramovitch et al, 1998a). Moreover, we showed that the presence of microtumours in an injured tissue inhibited the healing process, leaving an open persistent wound. O'Reilly et al (1994, 1996) showed recently that excision of a primary tumour accelerates the growth of a secondary tumour and metastases in other organs. This acceleration of tumour growth has been attributed to the removal of primary tumour-generated inhibitory factors. However, recent studies have shown that surgical wounding of normal tissues significantly stimulated the growth of malignant tissues without the concomitant excision of a tumour mass (Bogden et al, 1997). This humoral stimulating effect was not histologically specific or species specific (Bogden et al, 1997).

The process of healing and repair of damaged tissue is highly regulated by a large number of specific soluble growth factors which are released within the wound environment and which appear to induce neovascularization, inflammation, cell proliferation and deposition of collagen and other extracellular matrix molecules within the wound. These events are stimulated and regulated by mitogens and chemotactic factors which are secreted from cells in the wound border and from inflammatory cells (Lynch, 1991; Frank et al, 1995; Moulin, 1995). It appears that the same mediators of cell growth and stromal synthesis are involved in malignancy, fetal growth and wound healing. Many growth factors are released during tissue repair, and some of them such as fibroblast growth factors (FGFs), transforming growth factor α (TGF-α), epidermal growth factor (EGF), platelet-derived growth factor (PDGF), vascular endothelial growth factor (VEGF), tumour necrosis factor α (TNF-α), TGF-β and heparin-binding epidermal growth factor-like growth factor (HB-EGF) have been shown to be angiogenic in vivo (Lynch, 1991; Moulin, 1995; Abramovitch et al, 1998b). In addition, many of these growth factors are known to be directly mitogenic to tumour cells. It was shown previously that EGF, PDGF-BB and other growth factors affect glioma spheroids growth, migration and invasion (Pedersen et al, 1994). An additional enhancing mechanism could be the induction of VEGF secretion in the tumour as a result of exposure to growth factors released from the wound. It was recently shown

*Received 16 October 1997*
*Revised 6 May 1998*
*Accepted 12 June 1998*

*Correspondence to:* M Neeman

*Current address: Department of Animal Sciences, Faculty of Agriculture, Hebrew University, Rehovot, Israel

†The first two authors contributed equally to this work that VEGF secretion was induced in glioma cells by physiological concentrations of EGF, PDGF-BB and basic fibroblast growth factor (bFGF) (Tsai et al, 1995).

In light of the complexity of wound healing and the multiple cell types and growth factors involved, it is of interest to determine whether the stimulatory effects of injuries on tumour progression can be attributed to a restricted repertoire of molecules whose activity could possibly be antagonized.

The goal of this study was to identify the key growth factors that are found in healing wounds and exert a major stimulatory effect on tumour growth. In order to study the molecular basis for this stimulatory effect, we developed an experimental model using wound fluid derived from a cutaneous wound in pig (Marikovsky et al, 1993) and injected locally to an implanted C6 glioma spheroid. Using this system, we could reproduce the stimulatory effects of the wound on tumour progression, and could analyse the active constituents present in the wound. We show here that wound fluid collected during the first 3 days of pig injury enhances tumour growth in vivo, that wound fluid is angiogenic in vivo and mitogenic to bovine endothelial cells in vitro, and that wound fluid is directly mitogenic to C6 glioma cells. HB-EGF and PDGF were previously shown to be the major heparin-binding growth factors present in wound fluid (Marikovsky et al, 1993). Moreover, we have recently demonstrated that HB-EGF is angiogenic in vivo and induces VEGF expression in vascular smooth muscle cells (Abramovitch et al, 1998*b*). Here, we demonstrate that in wound fluid HB-EGF and PDGF are the major mitogens for C6 glioma cells.

### MATERIALS AND METHODS

### Materials

Human PDGF-BB and neutralizing anti-human PDGF antibodies were purchased from Collaborative Biomedical Products (Bedford, MA, USA). Recombinant HB-EGF and neutralizing anti-human HB-EGF polyclonal antibody 197 were kindly provided by Dr Judith A Abraham (Scios Nova, Mountain View, CA, USA). Neutralizing anti-HB-EGF polyclonal antibody 197 was raised in goats, directed against recombinant 77-amino-acid human HB-EGF. Antibodies were incubated with the wound fluid for 16 h (20 μg ml$^{-1}$, 4°C) and then added to cells. The antibodies are specific and do not interact with either EGF, TGF-α or amphiregulin. Pig plasma (Sigma) was used as a control for the effect of wound fluid.

### Cell culture and spheroid preparation

C6 rat glioma cells were routinely cultured in DMEM (Dulbecco's modified Eagle medium) supplemented with 5% fetal calf serum (FCS, Biological Industries, Israel), penicillin (50 unit ml$^{-1}$), streptomycin (50 μg ml$^{-1}$) and fungizone (125 μg ml$^{-1}$) (Biolab). Aggregation of cells into small spheroids of about 150 μm was initiated in agar-coated bacteriological plates. After 4–5 days in culture, the spheroid suspension was transferred to a 250-ml spinner flask (Bellco, USA) and the medium changed every other day for approximately 6 weeks. Other parameters of spheroid culture were as reported previously (Abramovitch et al, 1995, 1998*c*).

### Spheroid growth kinetics

Spheroids used for growth measurements were cultured individually in 24-well plates (Nunc, Denmark) coated with agar. An overlay suspension of 0.5 ml DMEM with the corresponding growth factor (1 ng ml$^{-1}$) was added. Spheroid diameter was measured daily using a microscope with a calibrated reticle in the eyepiece. The spheroid volume was calculated from the diameter measurements (four spheroids per treatment).

### Spheroid implantation in nude mice

Male CD1-nude mice (2 months old, 30 g body weight) were anaesthetized with a single dose of 75 μg g$^{-1}$ ketamine plus 3 μg g$^{-1}$ xylazine (i.p.) and placed in a sterile laminar flow hood. A single spheroid per mouse (approximately 1 mm in diameter) was implanted subcutaneously in the lower back through a 4-mm incision as reported previously (Abramovitch et al, 1995). The incision was formed by fine surgical scissors and closed with cyanoacrylate (Super Glue-3, Loctite, Ireland).

### Wound fluid preparation

Wound fluid (WF) was derived from injured pigs at various days after injury as reported previously (Marikovsky et al, 1993). Briefly, medium partial-thickness excisional wounds (15 × 15 × 1.2 mm) were created on the back of female Large White × Landraei pigs (40 kg). Each wound was covered with a liquid-tight vinyl chamber filled with 1.2 ml of normal saline containing penicillin (100 units ml$^{-1}$) and streptomycin sulphate (100 μg ml$^{-1}$). The solution from all wounds was pooled daily after injury, centrifuged, filtered through 0.45-μm filters and frozen at –20°C. Control solutions consisted of saline, penicillin (100 units ml$^{-1}$) and streptomycin sulphate (100 μg ml$^{-1}$).

### Wound fluid injections to tumour-bearing mice

WF derived from injured pigs (first 2 days after injury) was injected in mice in which a spheroid was implanted more than 1 cm away from the incision (subcutaneous injections began with spheroid implantation and continued every 2–3 days, close to the implanted spheroid). Control animals were injected with saline or with phosphate-buffered saline (PBS) supplemented with antibiotics or with pig plasma.

### Analysis of the angiogenic activity of wound fluid

Spherical agarose beads of approximately 1 mm in diameter were formed from 4% low gelling temperature agarose (Sigma) with either phosphate-buffered saline (PBS) or basic fibroblast growth factor (b-FGF; 0.5 or 5 μg), pig plasma or WF (fourfold concentrated). The tested compounds (5 μl per bead) were warmed to 38°C by placing them for a few seconds in sterile micro test tubes in a dry bath. Agarose solution (6% in saline, 45°C) was then added (10 μl per bead), and beads were formed above ice using a 20-μl pipette tip. Beads were implanted in nude mice 1 cm from the incision site, one bead per mouse. All control beads contained antibiotics as in the wound fluid (penicillin, 100 units ml$^{-1}$ and streptomycin sulphate, 100 μg ml$^{-1}$).

© Cancer Research Campaign 1999

### Magnetic resonance imaging (MRI) of the implanted spheroids or beads

MRI experiments were performed on a horizontal 4.7 T Bruker-Biospec spectrometer using a 2-cm surface coil. Mice were anaesthetized with a single dose of 75 µg g$^{-1}$ ketamine plus 3 µg g$^{-1}$ xylazine (i.p.), and placed supine with the tumour located at the centre of the surface coil. Gradient echo images (slice thickness of 0.5–0.6 mm, TR 100 ms, 256 × 256 pixels, in plane resolution of 110 µm) were acquired with echo time of 20 ms. Growth of the capillary bed was reflected by reduction of the mean intensity at a region of interest of 1 mm surrounding the spheroid or the agarose bead (Abramovitch et al, 1995, 1998*a,c*; Schiffenbauer et al, 1997). Data is reported here as the apparent vessel density [$AVD_{MRI} = -\ln S(a)/S(0)$], in which $S$(a) is the mean intensity at a region of interest of 1 mm surrounding the spheroid or the agarose bead and $S$(0) is the mean intensity of a distant muscle. Angiogenic capacity of the implanted beads was evaluated from the difference between $AVD_{MRI}$ on day 4 and $AVD_{MRI}$ on day 1 after implantation:

$$\Delta_{AVD} = AVD_{MRI}(\text{day 4}) - AVD_{MRI}(\text{day 1})$$

Tumour volume was determined from two orthogonal sets of multislice gradient echo images covering the entire tumour, as reported previously (Abramovitch et al, 1995, 1998*a*).

MRI data was analysed on a Personal Iris work station (Silicon Graphics, USA) with software from NMRi (Tripos). Statistical significance of treatments was determined using Student's *t*-test or ANOVA. Errors reported are the standard deviation.

### Measurement of DNA synthesis

C6 rat glioma cells were plated in 96-well plates (Nunc, Denmark) (5000 cells per well) in DMEM with 5% FCS. After 6 h, the cells were rinsed and incubated for 48 h in serum-free medium. Wound fluid, pig plasma or growth factors were then added for 24 h (triplicates). [$^3$H]thymidine 5 µCi ml$^{-1}$ (Rotem Industries, Israel) was added to the cells for the last 14 h (10 µl). The cells were rinsed with 100 µl methanol for 10 min, followed by 200 µl 5% cold trichloroacetic acid. Afterwards, cells were washed with water and lysed with 150 µl 0.5 M sodium hydroxide. Radioactive thymidine incorporation into DNA was determined for 60 s with 3 ml scintillation liquid (Ultima Gold, Packard) in a liquid scintillation β-counter.

Brain bovine capillary endothelial cells (BBCE) were plated in 24-well plates (6000 cells per well) in 500 µl medium [low glucose DMEM (1 g l$^{-1}$) +10% Colorado calf serum (CS) (Gibco, USA) and antibiotics: penicillin (100 units ml$^{-1}$), streptomycin (100 µg ml$^{-1}$), 2 mM glutamine (Biolab, Israel) (PSG).

After 24 h, the medium was changed to serum starvation medium (2% CS, 0.5% BSA, and PSG) for 24 h in the presence of wound fluid or wound fluid-derived growth factors. [$^3$H]thymidine (0.6 µCi) was added for the last 6 h. DNA synthesis assays (duplicates) were performed as described above.

All values were scaled by the counts from the respective control samples that were exposed to serum starvation medium alone.

### Histology

Skin with tumour specimens were fixed in neutral buffer formaldehyde (pH = 7) for 24 h, washed in 70% ethanol, embedded in paraffin, sectioned and stained with light green (Masson), eosin and haematoxylin.

### RESULTS

### Exogenous wound fluid promotes tumour neovascularization and growth

The stimulatory effects of wounds on tumour growth are clearly evident for in situ wounds on C6 glioma tumours implanted in nude mice (Abramovitch et al, 1998*a*). Unfortunately, the minute amounts of growth factors released in these in situ wounds do not allow for molecular analysis of the role of the different growth factors. To elucidate this point, we resorted to a more complex experimental system. The stimulatory effect of the wound on tumour progression was studied here using wound fluid (WF) derived from a pig wound model. The first step in this project was to test whether WF stimulated vascularization of implanted spheroids and accelerated tumour growth, as observed previously for in situ dermal incisions (Abramovitch et al, 1998*a*). Wound fluid was derived from partial thickness excisional wounds created on the back of a pig as reported previously (Marikovsky et al, 1993), and was injected subcutaneously next to an implanted C6 glioma spheroid (Figure 1). Control mice were injected with PBS ($n = 9$) or with pig plasma ($n = 2$). Wound fluid administration was initiated

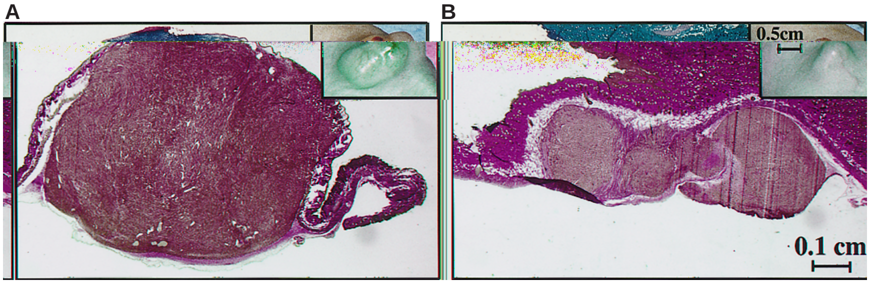

**Figure 1** Wound fluid enhances tumour growth for C6 glioma spheroids implanted in nude mice. (**A** and **B**) Histological sections of C6 glioma tumours 21 days after implantation (bar, 0.1 cm). Mice were injected with a total amount of 2.4 ml of either wound fluid derived from day 2 of pig injury (**A**) or PBS (**B**); subcutaneous injections, close to the implanted spheroid, eight injections of 300 µl each every 2–3 days starting on the day of spheroid implantation. Tumour treated with wound fluid showed a significant effect through the entire experiment and tumour volume was fourfold larger at the end of the experiment. Inserts, photographs of the same tumours as in **A** and **B** (bar, 0.5 cm)

 *© Cancer Research Campaign 1999*

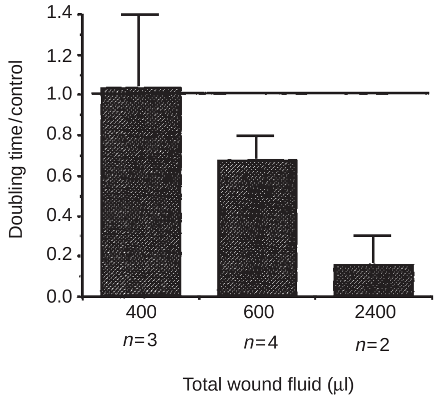

**Figure 2** The effect of wound fluid derived from pig on the progression of C6 glioma tumours in nude mice. Tumour volume was determined every 2–3 days by gradient echo MRI from two orthogonal sets of multislice images covering the entire tumour (Bruker Biospec spectrometer, 4.7 T; TE = 20 ms, TR = 100 ms, 0.11 mm in plane resolution; 0.5–0.6 mm slice thickness). Wound fluid reduced tumour doubling time for C6 glioma tumours in vivo in a dose-dependent manner on the 3rd week after implantation. WF derived from injured pigs (days 1–2) was injected into the mice subcutaneously, close to the implanted spheroid, every 2–3 days. Control animals were injected with saline or pig plasma

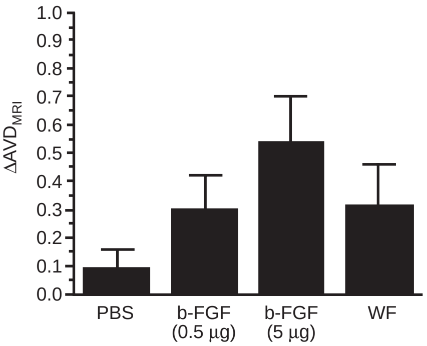

**Figure 3** WF promotes neovascularization in vivo in beads implanted subcutaneously in nude mice. Agarose beads containing the various materials were implanted as described in Materials and methods. The angiogenic potential of WF in vivo is demonstrated quantitatively by MRI. The apparent vessel density ($AVD_{MRI}$) was determined from the degree of signal loss in gradient echo MRI, as described previously (Abramovitch et al, 1995, 1998*a*; Schiffenbauer et al, 1997). Neovascularization around the beads was quantitatively evaluated by subtraction of the $AVD_{MRI}$ measured on day 1 from the one measured on day 4 after implantation ($\triangle AVD_{MRI}$). PBS, as a negative control, did not induce neovascularization around the bead ($n$ = 8), whereas bFGF, as a positive control, caused significant neovascularization around the beads ($n$ = 3 and $n$ = 7 for 0.5 and 5 µg bFGF respectively). In comparison, wound fluid (WF; 5 µl) from the first 3 days after injury induced significant neovascularization around the beads ($n$ = 6)

at the time of spheroid implantation. Care was taken to position the spheroid at a distance larger than 1 cm from the incision so as to avoid direct stimulation of tumour growth by the in situ surgical wound (Abramovitch et al, 1998*a*).

As observed previously for spheroids implanted on a full thickness dermal incision, treatment with WF reduced the lag in tumour growth (from 5 days to 3 days; $n$ = 5), and the early stages of neovascularization were enhanced (vascular density was maximal on day 4, relative to day 6 for control spheroids; $n$ = 5). In contrast to wound fluid, pig plasma and PBS did not induce tumour growth. The apparent vessel density ($AVD_{MRI}$) was derived from gradient echo images of tumour treated with WF (6 injections, 100 µl each, every 48 h) 8 days after implantation. Vessel density was much larger in WF-treated spheroids than that observed in control spheroids (0.964 ± 0.19, $n$ = 5, and 0.447 ± 0.19, $n$ = 8, respectively; $P$ = 0.0003). Histological sections show that the enhanced rate of tumour growth can be attributed to the tumour cells themselves and not to infiltrating host cells such as fibroblasts (Figure 1).

In addition to the proangiogenic activity, WF also accelerated the rate of tumour growth. Tumour growth in WF-treated mice was monitored by MRI and was compared with the rate of tumour growth in control mice. For mice that were injected with a total amount of 0.4 ml wound fluid (four injections of 100 µl WF each, every 2–3 days, $n$ = 3), the effect on tumour growth rate relative to control mice (four injections of 100 µl saline each, every 2–3 days, $n$ = 3) was evident during the first 4 days after spheroid implantation, but there was no observable effect on tumour development in the 3rd week of tumour growth (days 14–18) (Figure 2). For mice that were injected with a total amount of 0.6 ml wound fluid (six injections of 100 µl WF each, every 2–3 days, $n$ = 4), there was a 40% increase in growth rate (reduced doubling time) relative to control mice (six injections of 100 µl saline each, every 2–3 days, $n$ = 4) observable in the 3rd week (days 14–18) (Figure 2). Mice injected with a higher dose of WF, i.e. a total amount of 2.4 ml (eight injections of 300 µl WF, $n$ = 2), showed a significant effect relative to control mice (eight injections of 300 µl PBS, $n$ = 2, or 300 µl pig plasma, $n$ = 2) through the entire experiment (3 weeks). Tumour doubling time in WF-treated mice was 6.7 times faster than control saline or plasma-treated mice on days 14–18 (Figure 2), and tumour volume measured by MRI was fourfold larger. The maximal volume of WF (2.4 ml) is the amount of WF released during 1 day from a 2.5 $cm^2$ partial thickness excisional wound in our experimental wound model in pig. These experiments suggest that the duration and dose of cumulative exposure to wound-derived factors affects the degree of tumour stimulation.

### Wound fluid is angiogenic

The angiogenic capacity of WF was assayed in vivo by MRI. Agarose beads containing PBS, b-FGF or WF derived 1 day after injury were implanted subcutaneously in nude mice as reported

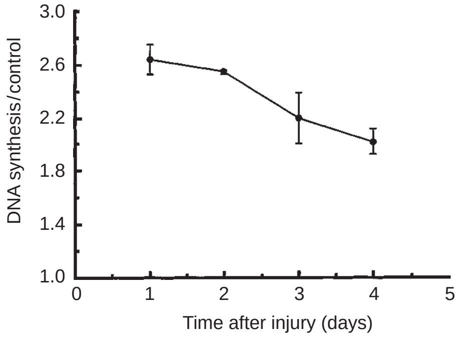

**Figure 4** Wound fluid increases DNA synthesis in endothelial cells. The angiogenic potential of wound fluid in vitro was tested by [$^3$H]thymidine incorporation into brain bovine capillary endothelial cells (BBCE) as described in Materials and methods. The angiogenic capacity of WF was maximal with WF derived 1 day after injury, and decreased slowly during the wound healing process

© Cancer Research Campaign 1999

previously (Abramovitch et al, 1995, 1998*a*). PBS, a negative control, did not cause neovascularization around the bead ($\Delta_{AVD}$ = 0.08 ± 0.06; *n* = 8), whereas b-FGF, a known angiogenic growth factor, showed dose-dependent increase in the AVD (Figure 3). WF derived from injured pigs during the first 3 days after injury (fourfold concentrated) showed significant neovascularization around the beads ($\Delta_{AVD}$ = 0.32 ± 0.14; *n* = 6), which was comparable to that observed for 0.5 μg b-FGF ($\Delta_{AVD}$ = 0.3 ± 0.11; *n* = 3) and less than that of 5 μg b-FGF ($\Delta_{AVD}$ = 0.54 ± 0.2; *n* = 7) (Figure 3). The specificity of the effect of pig-derived WF was evaluated by comparison with pig plasma. In contrast to WF, implantation of an agarose bead containing pig plasma (*n* = 4) did not cause neovascularization around the bead.

In accord with the angiogenic capacity of WF in vivo, WF was mitogenic to endothelial cells in vitro (Figure 4). WF derived 1 day after pig injury showed the maximal enhancement of DNA synthesis ([$^3$H]thymidine incorporation) in bovine endothelial cells: 2.6-fold increase relative to control cells (Figure 4). This effect slowly decreased to twofold for WF derived 4 days after injury.

### Wound fluid enhances growth of C6 glioma in vitro

The stimulatory effect of WF on the rate of tumour growth suggests that, in addition to its angiogenic activity, WF possibly also exerts a direct effect on the rate of tumour cell proliferation. To identify possible growth factors in WF which promote proliferation of C6 glioma cells, we studied the direct effect of growth factors known to be present in WF. Basic-FGF, HB-EGF and PDGF enhanced DNA synthesis in C6 glioma cells, as measured by thymidine incorporation, in monolayer cell culture (Figure 5A). Basic-FGF increased DNA synthesis by approximately 1.9-fold above control. The maximal effect was obtained at a concentration of 1 ng ml$^{-1}$. HB-EGF increased DNA synthesis by 1.95-fold over negative control, the maximal effect being obtained by 3.3 ng ml$^{-1}$. PDGF increased DNA synthesis by 1.74-fold above control and the maximal effect was obtained by 3.3 ng ml$^{-1}$.

The effect of these growth factors on the growth of C6 glioma in three-dimensional spheroid culture was also studied (Figure 5B). Spheroid growth was significantly stimulated by 1 ng ml$^{-1}$ of PDGF, EGF and HB-EGF (2.21- to 2.36-fold increased volume in 4 days) compared with control (1.49-fold increased volume) (Figure 5B). Basic-FGF had a, relatively, smaller effect on spheroid growth (1.86-fold).

In view of the direct mitogenic activity of PDGF, EGF, HB-EGF and bFGF, we checked the effect of WF on C6 glioma cells. As expected, WF was indeed directly mitogenic to the C6 glioma tumour cells (Figure 6A). WF derived from the first 3 days after injury enhanced DNA synthesis in C6 glioma cells in a dose-dependent manner. WF from days 1–2 after injury increased DNA

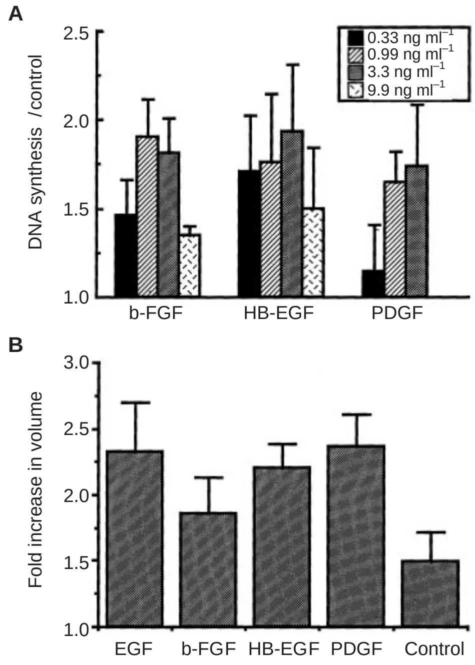

**Figure 5** Growth factors associated with wound repair are mitogenic to C6 glioma. The mitogenic effect of growth factors was determined in vitro. (**A**) Basic-FGF, HB-EGF and PDGF, growth factors present in wound fluid during days 1–2, enhanced DNA synthesis in C6 glioma cells. (**B**) Effect of growth factors on spheroid growth. PDGF, EGF and HB-EGF at 1 ng ml$^{-1}$ significantly enhanced C6 glioma spheroid growth in vitro. Spheroids were cultured individually on agar as described in Materials and methods. For each spheroid, the gain in volume after 4 days in culture was calculated (four spheroids per treatment)

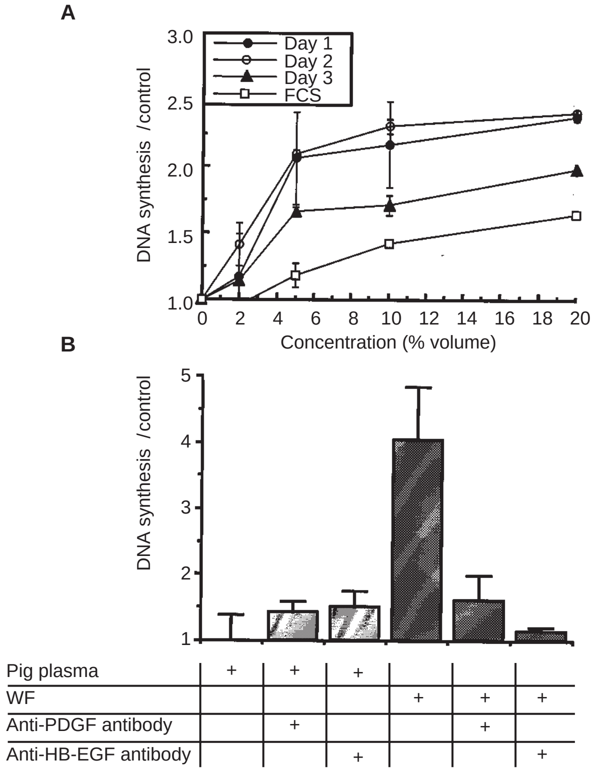

**Figure 6** WF is mitogenic to C6 glioma cells. (**A**) The mitogenic activity of WF on C6 glioma cells in vitro was determined by [$^3$H]thymidine incorporation. WF derived from the first 3 days after injury enhanced DNA synthesis in a dose-dependent manner. (**B**) HB-EGF and PDGF are the main mitogens present in WF for C6 glioma cells. Neutralizing antibodies against either PDGF-BB or against HB-EGF blocked most of the enhancing effect of wound fluid derived 2 days after pig injury. Pig plasma had no effect on DNA synthesis in C6 glioma cells

*© Cancer Research Campaign 1999*

synthesis by approximately 2.4-fold above control. WF derived 3 days after injury increased DNA synthesis by twofold. WF was more mitogenic to C6 glioma than FCS. Pig plasma (10%), in contrast, had no effect on thymidine incorporation in C6 glioma cells (Figure 6B).

With the demonstration that growth factors known to be present in WF can stimulate tumour cell proliferation, it was of interest to evaluate their role in the mitogenicity of WF for C6 glioma cells. This was carried out by antagonizing their activity in WF with neutralizing antibodies. Neutralizing antibodies against either PDGF-BB or HB-EGF blocked most of the mitogenic activity of WF derived 2 days after pig injury for C6 glioma in vitro (Figure 6B). WF derived 2 days after pig injury increased DNA synthesis in C6 glioma cells fourfold above control. Neutralizing antibodies against HB-EGF suppressed 95% of the mitogenic effect of WF and neutralizing antibodies against PDGF-BB suppressed 80% of the mitogenic effect of WF. Thus, a significant contribution to the direct mitogenic activity of WF on the tumour cells can be attributed to these two growth factors. In contrast to WF, pig plasma had no effect on DNA synthesis in C6 glioma cells (Figure 6B).

### DISCUSSION

The increased probability of recurrence and accelerated tumour growth in the location of tissue injury are common clinical complications associated with the invasive procedures frequently used in cancer therapy. Understanding the fundamental principles that are involved may provide a way to possibly reduce the incidence and extent of tumour induction resulting from treatment. We have previously demonstrated that MRI can be used to follow primary tumour angiogenesis in vivo in a model system of an implanted multicellular spheroid (Abramovitch et al, 1995), as well as for monitoring the induction of spheroid neovascularization by proximal injury (Abramovitch et al, 1998*a*). In the study reported here, we applied quantitative MRI to measure the angiogenic properties of wound fluid as well as the stimulatory effect of wound fluid on the progression of implanted C6 glioma spheroids in vivo.

The phenomenon of tumour growth enhancement by injury was reproduced by injecting wound fluid (derived from injured pigs 1–2 days after wound formation) to tumour-bearing mice. Wound fluid stimulated DNA synthesis in endothelial cells, and accordingly increased vascularization in mouse skin by implanted agarose beads containing wound fluid. In addition, wound fluid showed direct mitogenic activity on C6 glioma cells. Thus, this approach made it possible to identify the growth factors which are known to participate in wound healing for their role and importance in the enhancement of tumour proliferation and vascularization resulting from injury. HB-EGF is a well-characterized 22-kDa glycoprotein that binds the EGF receptor with high affinity and is mitogenic for fibroblasts, smooth muscle cells and epithelial cells (Higashiyama et al, 1991; Marikovsky et al, 1993). Both HB-EGF and PDGF were previously shown to be present in wound fluid (Marikovsky et al, 1993). Moreover, HB-EGF was shown to be the most prominent heparin-binding mitogen for fibroblasts (Marikovsky et al, 1993) and for epidermal keratinocytes (Marikovsky et al, 1996) during wound repair. Recently, we showed that HB-EGF is angiogenic in vivo, an activity which was attributed to expression and secretion of VEGF by HB-EGF-stimulated vascular smooth muscle cells (Abramovitch et al, 1998*b*).

In the study presented here, HB-EGF and PDGF were found to be the major mitogens for C6 glioma cells present in wound fluid. We suggest, therefore, that HB-EGF and PDGF are likely candidates for mediating the direct stimulatory effect of wounds on tumour progression. The fact that neutralization of either HB-EGF or PDGF inhibited almost all the induced mitogenic effect of wound fluid on C6 cells is in accord with the findings of a synergistic relation between these two growth factors (M Marikovsky and M Klagsbrun, unpublished data). A similar induction of proliferation of breast and ovarian cancer cells was recently reported for HB-EGF released in tumours by infiltrating T-cells (Peoples et al, 1995). In addition, it was shown previously that EGF, PDGF-BB and other growth factors affect glioma spheroids growth, migration and invasion (Pedersen et al, 1994). The important effect of PDGF is reinforced because it was shown that high-grade primary gliomas express increased levels of PDGF receptors, and the presence of PDGF receptors appears to correlate with tumour progression (Strawn et al, 1994). By using dominant negative PDGF-β receptors, growth of C6 glioma could be inhibited both in vitro and in vivo. Glioma progression has also been correlated with amplification of the EGF receptor gene (Libermann et al, 1985).

The study reported here suggests that specific inhibition of the key growth factors in wound fluid could possibly be used to protect cancer patients undergoing surgical intervention from the tumour stimulatory effects of injury. However, the effect of neutralizing the activity of these growth factors, known to be important in wound healing, must first be carefully evaluated. Furthermore, the specific repertoire of dominant growth factors could be tumour specific and might be different for rat and human tumours, as reported previously for glioma cells lines (Pollack et al, 1991).

In summary, we demonstrate here that growth factors released in injury sites are directly mitogenic to tumour cells, leading to accelerated tumour growth. For C6 glioma cells, the major mitogenic activity was attributed to HB-EGF and PDGF. In addition, wound fluid contributes a significant angiogenic activity and thus could enhance the angiogenic switch of avascular, dormant microtumours. Appropriate inhibition of these stimulatory pathways may improve the recovery from surgery and prognosis in cancer patients.

### ACKNOWLEDGEMENTS

Basic-FGF was kindly provided by Professor Gera Neufeld (Technion, Israel). Bovine endothelial cells were kindly provided by Professor Israel Vlodavsky (Hadassah Medical Center, Israel). This work was supported by a Research Career Development Award from the Israel Cancer Research Fund and by the Israel Science Foundation founded by the Israel Academy of Sciences and Humanities (to MN). MN is incumbent of Dr Phil Gold Career Development Chair in Cancer Research. RA is a recipient of a fellowship from the Charles Clore foundation.

### REFERENCES

Abramovitch R, Meir G and Neeman M (1995) Neovascularization induced growth of implanted C6 glioma multicellular spheroids: magnetic resonance microimaging. *Cancer Res* **55**: 1956–1962

Abramovitch R, Marikovsky M, Meir G and Neeman M (1998*a*) Stimulation of tumour angiogenesis by proximal wounds: spatial and temporal analysis by MRI. *Br J Cancer* **77**: 440–447

© Cancer Research Campaign 1999

Abramovitch R, Neeman M, Reich R, Stein I, Keshet E, Abraham J, Solomon A and Marikovsky M (1998*b*). Intercellular communication between vascular smooth muscle and endothelial cells mediated by HB-EGF and VEGF. *FEBS Lett* **425**: 441–447

Abramovitch R, Frenkiel D and Neeman M (1998*c*) Analysis of subcutaneous angiogenesis by gradient echo magnetic resonance imaging. *Magn Reson Med* **39**: 813–824

Bogden AE, Moreau JP and Eden PA (1997) Proliferative response of human and animal tumours to surgical wounding of normal tissues: onset, duration and inhibition. *Br J Cancer* **75**: 1021–1027

Deelman HT (1927) The part played by injury and repair in the development of cancer. *Br Med J* **1**: 872

Dvorak HF, Brown LF, Detmar M and Dvorak AM (1995) Vascular permeability factor/vascular endothelial growth factor, microvascular hyperpermeability, and angiogenesis. *Am J Pathol* **146**: 1029–1039

Frank S, Hubner G, Breier G, Longaker MT, Greenhalgh DG and Werner S (1995) Regulation of vascular endothelial growth factor expression in cultured keratinocytes. Implications for normal and impaired wound healing. *J Biol Chem* **270**: 12607–12613

Higashiyama S, Abraham JA, Miller J, Fiddes JC and Klagsbrun M (1991) A heparin-binding growth factor secreted by macrophage-like cells that is related to EGF. *Science* **251**: 936–939

Libermann TA, Nusbaum HR, Razon N, Kris R, Lax I, Soreq H, Whittle N, Waterfield MD, Ullrich A and Schlessinger J (1985) Amplification and overexpression of the EGF receptor gene in primary human glioblastomas. *Cell Sci* **3**(suppl): 161–172

Lynch SE (1991) Interactions of growth factors in tissue repair. *Prog Clin Biol Res* **365**: 341–357

Marikovsky M, Breuing K, Liu PY, Eriksson E, Higashiyama S, Farber P, Abraham J and Klagsbrun M (1993) Appearance of heparin-binding EGF-like growth factor in wound fluid as a response to injury. *Proc Natl Acad Sci USA* **90**: 3889–3893

Marikovsky M, Vogt P, Eriksson E, Rubin JS, Taylor WG, Joachim S and Klagsbrun M (1996) Wound fluid-derived heparin-binding EGF-like growth factor (HB-EGF) is synergistic with insulin-like growth factor-I for Balb/MK keratinocyte proliferation. *J Invest Dermatol* **106**: 616–621

Moulin V (1995) Growth factors in skin wound healing. *Eur J Cell Biol* **68**: 1–7

Murthy SM, Goldschmidt RA, Rao LN, Ammirati M, Buchmann T and Scanlon EF (1989) The influence of surgical trauma on experimental metastasis. *Cancer* **64**: 2035–2044

Murthy MS, Summaria LJ, Miller RJ, Wyse TB, Goldschmidt RA and Scanlon EF (1991) Inhibition of tumor implantation at sites of trauma by plasminogen activators. *Cancer* **68**: 1724–1730

O'Reilly MS, Holmgren L, Shing Y, Chen C, Rosenthal RA, Moses M, Lane WS, Cao Y, Sage EH and Folkman J (1994) Angiostatin: a novel angiogenesis inhibitor that mediates the suppression of metastases by a Lewis lung carcinoma. *Cell* **79**: 315–328

O'Reilly MS, Holmgren L, Chen C and Folkman J (1996) Angiostatin induces and sustains dormancy of human primary tumors in mice. *Nature Med* **2**: 689–692

Pedersen PH, Ness GO, Engebraaten O, Bjerkvig R, Lillehaug JR and Laerum OD (1994) Heterogeneous response to the growth factors [EGF, PDGF (bb), TGF-alpha, bFGF, IL-2] on glioma spheroid growth, migration and invasion. *Int J Cancer* **56**: 255–261

Peoples GE, Blotnick S, Takahashi K, Freeman MR, Klagsbrun M and Eberlein TJ (1995) T lymphocytes that infiltrate tumors and atherosclerotic plaques produce heparin-binding epidermal growth factor-like growth factor and basic fibroblast growth factor: a potential pathologic role. *Proc Natl Acad Sci USA* **92**: 6547–6251

Pollack IF, Randall MS, Kristofik MP, Kelly RH, Selker RG and Vertosick Jr FT (1991) Response of low-passage human malignant gliomas in vitro to stimulation and selective inhibition of growth factor-mediated pathways. *J Neurosurg* **75**: 284–293

Schiffenbauer YS, Abramovitch R, Meir G, Nevo N, Holzinger M, Itin A, Keshet E and Neeman M (1997) Loss of ovarian function promotes angiogenesis in human ovarian carcinoma. *Proc Natl Acad Sci USA* **94**: 13203–13208

Strawn LM, Mann E, Elliger SS, Chu LM, Germain LL, Niederfellner G, Ullrich A and Shawver LK (1994) Inhibition of glioma cell growth by a truncated platelet-derived growth factor-beta receptor. *J Biol Chem* **269**: 21215–21222

Tsai JC, Goldman CK and Gillespie GY (1995) Vascular endothelial growth factor in human glioma cell lines: induced secretion by EGF, PDGF-BB, and bFGF. *J Neurosurg* **82**: 864–873

*© Cancer Research Campaign 1999*
